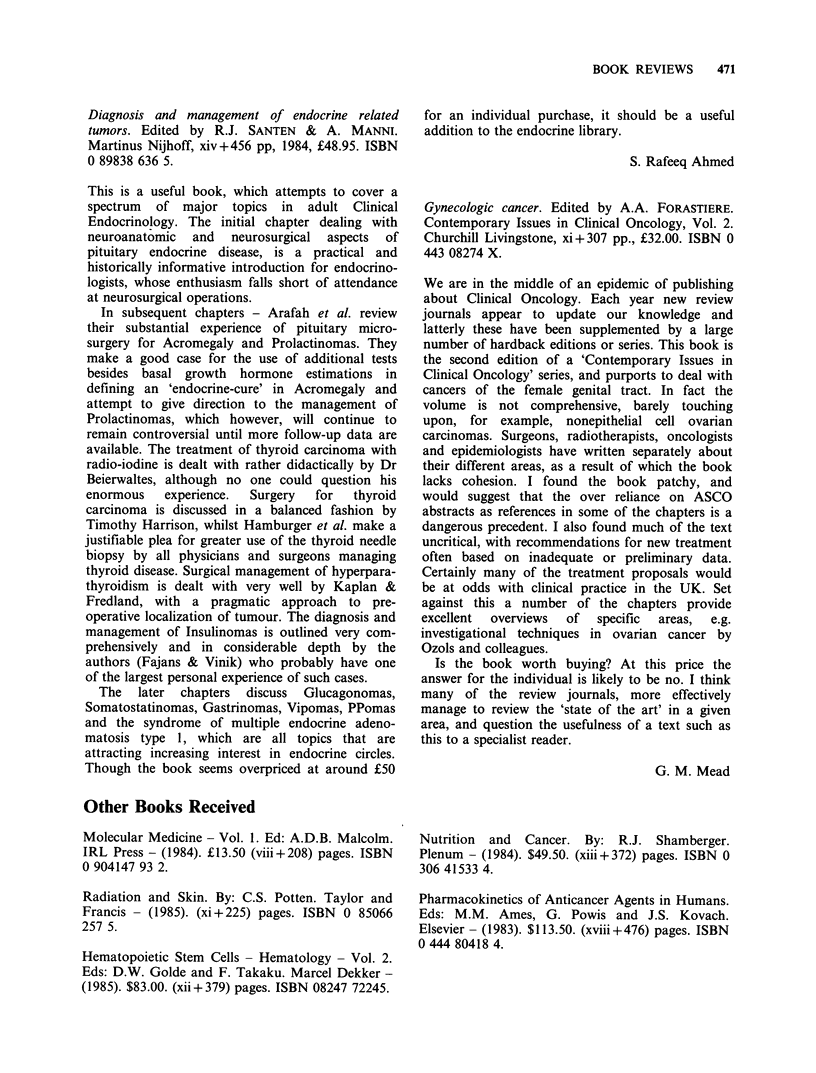# Gynecologic cancer

**Published:** 1985-09

**Authors:** G. M. Mead


*Gynecologic cancer*. Edited by A.A. FORASTIERE. Contemporary Issues in Clinical Oncology, Vol. 2. Churchill Livingstone, xi+307 pp., £32.00. ISBN 0 443 08274 X.

We are in the middle of an epidemic of publishing about Clinical Oncology. Each year new review journals appear to update our knowledge and latterly these have been supplemented by a large number of hardback editions or series. This book is the second edition of a 'Contemporary Issues in Clinical Oncology' series, and purports to deal with cancers of the female genital tract. In fact the volume is not comprehensive, barely touching upon, for example, nonepithelial cell ovarian carcinomas. Surgeons, radiotherapists, oncologists and epidemiologists have written separately about their different areas, as a result of which the book lacks cohesion. I found the book patchy, and would suggest that the over reliance on ASCO abstracts as references in some of the chapters is a dangerous precedent. I also found much of the text uncritical, with recommendations for new treatment often based on inadequate or preliminary data. Certainly many of the treatment proposals would be at odds with clinical practice in the UK. Set against this a number of the chapters provide excellent overviews of specific areas, e.g. investigational techniques in ovarian cancer by Ozols and colleagues.

Is the book worth buying? At this price the answer for the individual is likely to be no. I think many of the review journals, more effectively manage to review the 'state of the art' in a given area, and question the usefulness of a text such as this to a specialist reader.

G. M. Mead